# Environmental harm from anaesthesia: the importance of clinical realism and chemical persistence: a reply

**DOI:** 10.1016/j.bjao.2025.100495

**Published:** 2025-10-10

**Authors:** Sarah-Louise Watson, Tom E.F. Abbott

**Affiliations:** 1 Barts and the London School of Medicine and Dentistry, Queen Mary University of London, London, UK; 2 Critical Care and Perioperative Medicine Research Group, William Harvey Research Institute, Queen Mary University of London, London, UK

**Keywords:** climate change, desflurane, environment, global warming, propofol, sevoflurane volatile anaesthesia

Editor

We thank Kalmar and colleagues^1^ for their letter in response to our review of the environmental impact of commonly used anaesthetic agents. Our aim was to report the environmental impact of i.v. and volatile anaesthetic agents using a cradle-to-grave lifecycle approach. Kalmar and colleagues^1^ were concerned with our focus on global warming over ecotoxicity and our framing of the environmental impact of TIVA *vs* volatile agents. We aim to address these below.

More than 5 million people undergo surgery in the UK each year, and this frequency is increasing.^2^ This means a large volume of anaesthetic agents are used each year and will be increasing in use; this will have a considerable effect on our environment. Kalmar and colleagues^1^ highlight the significant environmental hazard of volatile anaesthetics, which contribute to global warming and chemical pollution. Sevoflurane, desflurane, and isoflurane degrade to trifluoracetic acid (TFA) when released into the atmosphere and hexafluoroisopropanol (HFIP) when metabolised, which is excreted renally. These products are not biodegradable, bypass treatment plants, and persist in the environment. Kalmar and colleagues^1^ were concerned with the omission of persistent aquatic perfluoroalkyl and polyfluoroalkyl substances (PFAS) pollution from our review. It is a limitation of our review that our eligibility criteria meant that only papers including the cradle-to-grave lifecycle steps for anaesthetic agents were included. This created a narrow scope of papers that we could use and subsequently meant that papers reporting on the influence of anaesthetic agents on ecotoxicity and aquatic persistence were not included. A wider search criterion in our review, including phrases such as ecotoxicity, bioaccumulation, and aquatic persistence, would have returned papers investigating this element of environmental damage caused by anaesthesia. None of the studies that included cradle-to-grave lifecycle steps reported on the environmental impact of HFIP or TFA. This reflects the literature surrounding environmental research into anaesthesia; research tends to focus on greenhouse gas emissions rather than the broader environmental impact of PFAS agents.^3^ Consequently, there are limited data on the aquatic bioconcentration of HFIP. Our review relied on carbon dioxide equivalent (CO_2_e) metrics because the literature on environmental risks of anaesthesia focuses on greenhouse gas contribution rather than ecotoxicity and bioaccumulation. Further research is needed to investigate and understand the impact of volatile anaesthesia on ecotoxicity and aquatic accumulation.

Kalmar and colleagues^1^ highlight that propofol can be readily removed by ozonation in wastewater treatment plants, and that propofol metabolites degrade quickly in wastewater treatment plants. Unfortunately, ozonation is not widely used in the UK yet.^4^ In some cases, higher concentrations of propofol have been found to leave wastewater plants than entering, and some of this may be because of de-glucuronidation.^5^ Therefore, we could be underestimating the amount of propofol in the aquatic environment; propofol is not readily biodegradable.^6^ Propofol use in anaesthetics has increased as TIVA use increases; understanding its impact on the environment is non-negotiable even if there is a smaller impact in comparison with volatile anaesthetics.^7^

Regarding the concern in relation to our global surface temperature figure, this figure was provided by the Intergovernmental Panel on Climate Change (IPCC) in 2018, a United Nations body for assessing climate change. An updated report from the IPCC in 2023, when our review was written, stated that the global surface temperature was 0.95–1.20°C above pre-industrial concentrations between 2011 and 2020; this should have been updated in our review.^8^ There is some disagreement with global surface temperature increases, with the Copernicus climate change service reporting an increase of 1.48°C above the pre-industrial concentration between 1991 and 2020.^9^

We thank the authors for pointing out our wording with regard to the ratio of norketamine to ketamine in wastewater and the urine of patients administered ketamine. High concentrations of ketamine up to 10 μg L^–1^ have been found in hospital effluents.^10^ The study included in our review found that a ketamine concentration of 5 μg L^–1^ did cause a significant decrease in the number of live offspring of *Daphnia magna* compared with the control. This likely indicates some chronic toxicity in ketamine-containing effluents.^11^ As Kalmar and colleagues point out, ozonation is not regularly available yet; therefore, lidocaine and ketamine are still being released to the environment. Although we agree this poses less harm than volatile agents, it is still important to investigate and understand the environmental harm caused by these agents as there is room for improvement.

The authors highlight CO_2_e emissions generated from harvesting the soy oil needed for propofol is much less than the CO_2_ emissions generated from sevoflurane. Our aim was to report on the lifecycle domains of all anaesthetic agents, one of which was raw material for manufacturing. As such, we brought attention to the soy oil use in propofol, a raw material. Comparing this with the CO_2_ emissions generated by volatile anaesthetic use was out of the scope of our paper.

A limitation of our review is that there is a narrow focus on greenhouse gas emissions. This is primarily a result of our narrow scope, including only studies focusing on the cradle-to-grave lifecycle approach. This means we subsequently have not included further elements of environmental harm that volatile and i.v. anaesthetic agents can cause. This has been highlighted through the important work Kalmar and colleagues have previously published.^12^^,^^13^ Future work should extend to quantifying the impact of anaesthetic agents in terms of ecotoxicity, aquatic persistence, and bioaccumulative potential to better understand their impact on our environment. We agree that it is important to identify the most meaningful opportunities to reduce environmental harm, but all areas that can help to understand and reduce the impact of anaesthetic agents on the environment should be investigated.

## Declaration of interest

TA is supported by an NIHR Development and Skills Enhancement award; has received research funding from Barts Charity, the Academy of Medical Sciences, The Royal College of Anaesthetists, and the *British Journal of Anaesthesia*; has received honoraria from Merk and Edwards Life Sciences; and is Social Media Editor of the *British Journal of Anaesthesia*.
